# Assessment of animal owners’ compliance with short-course antibiotic treatment at the University of Gondar Veterinary Teaching Hospital, Northwest Ethiopia

**DOI:** 10.1186/s12917-026-05561-1

**Published:** 2026-05-18

**Authors:** Zenebe Jemere Aragaw, Samuel Derso Tezera, Asnakew Mulaw Berihun, Yesuneh Tefera Mekasha, Abibo Wondie Mekonen, Dejen Tekale Shiferaw, Achenef Melaku Beyene

**Affiliations:** 1 Department of Veterinary Clinical Medicine, College of Veterinary Medicine and Animal Sciences, University of Gondar, Gondar, Ethiopia; 2 Department of Pathobiology, College of Veterinary Medicine and Animal Sciences, University of Gondar, Gondar, Ethiopia; 3 Bioequivalence Center, Clinical Trial Directorate, Armauer Hansen Research Institute, Addis Ababa, Ethiopia; 4 Department of Veterinary Pharmacy, College of Veterinary Medicine and Animal Sciences, University of Gondar, Gondar, Ethiopia

**Keywords:** Animal owners, Antimicrobial stewardship, One health, Short-course antibiotic, Treatment compliance, University of Gondar Veterinary Teaching Hospital, Gondar, Ethiopia

## Abstract

**Background:**

Non-compliance with antibiotic treatment regimens can lead to treatment failure, antimicrobial resistance, compromised animal welfare, economic losses, and public health risks. Evidence on compliance among animal owners in Ethiopia is lacking, leaving a critical gap in understanding behaviors that affect antimicrobial stewardship and One Health strategies. This study provides the first evidence on compliance with short-course antibiotic treatments in Gondar Town, Ethiopia, by assessing factors that influence compliance, identifying reasons for non-compliance, and proposing evidence-based interventions.

**Methods:**

A prospective observational study was conducted at the University of Gondar Veterinary Teaching Hospital from February to August 2025. A total of 106 animal owners (27 cattle, 27 sheep, 26 dogs, and 26 horses) were enrolled. Compliance, defined as completion of all prescribed doses, was monitored through direct observation using follow-up checklists. Non-compliers were interviewed to capture reasons for missed treatments. Logistic regression analysis was performed using SPSS version 20 to identify predictors of compliance.

**Results:**

The overall compliance rate was 68.9%, indicating a moderate level of compliance. Compliance was significantly associated with education, farming experience, and prior experience with animal illness. Owners with primary (aOR = 6.526, *p* = 0.044) and tertiary educations (aOR = 7.256, *p* = 0.033), as well as those with five to ten years of farming experience (aOR = 13.193, *p* = 0.008), had markedly higher odds of following prescribed regimens. In contrast, owners encountering animal illness for the first time (aOR = 0.029, *p* = 0.005) were less likely to comply. The main reasons for non-compliance included premature discontinuation of treatment after perceived recovery (27.3%), forgetfulness and time constraints (27.3%).

**Conclusion:**

Animal owners showed moderate compliance, but gaps due to perceived recovery, forgetfulness, and time constraints persist. Tailored counseling for owners with limited illness experience and low literacy combined with peer-to-peer learning should be implemented.

**Supplementary Information:**

The online version contains supplementary material available at 10.1186/s12917-026-05561-1.

## Introduction

Treatment compliance refers to the accurate and consistent adherence to prescribed regimens, including correct dosage, duration, and timely administration of medications [1]. In veterinary medicine, animal owner compliance denotes the extent to which owners or caregivers follow instructions provided by veterinarians or animal healthcare providers [1, 2]. While “adherence” is increasingly used in human medicine to emphasize patient independence, “compliance” is more appropriate in veterinary contexts, where owners act as intermediaries in administering treatment and providing care for sick animals [1, 2]. Both compliance and adherence are often used interchangeably to describe how closely the actual use of a medication aligns with prescription instructions [3, 4].

Compliance matters because in antimicrobial therapy, strict adherence is essential to ensure treatment efficacy, safeguard animal welfare, and prevent the development of antimicrobial resistance (AMR) [1, 4]. Effective treatment outcomes depend on animal owners’ ability to follow veterinarian-recommended instructions, monitor responses, and report complications, which in turn relies on clear communication from veterinary professionals [5, 6]. Factors such as education, financial constraints, misperceptions, and regimen complexity (e.g., dosing frequency, duration) influence compliance [7, 8]. Methods to assess compliance include pill counts, self-reports, electronic monitoring, and follow-up observation [7].

Globally, compliance rates vary: Booth et al. [2] reported 56% in canine epilepsy; Adams et al. [7] found 97% for short-term antibiotic use in dogs, while Odom et al. [1] observed 47% of dog owners failing to follow instructions. Variation reflects owners’ prior experience and differences in monitoring periods [1, 2, 7]. Many owners discontinue treatment once clinical signs improve [9], overlooking that the primary goal of antimicrobial therapy is pathogen eradication rather than mere symptom relief [10]. This undermines disease management, prolongs illness, accelerates AMR, and increases costs [3, 11, 12].

Misunderstanding treatment goals, lack of AMR awareness, and confusion about side effects further reduce compliance [9, 13]. While global studies showed that compliance is shaped by many factors, considering the realities in Ethiopia context is essential to fully understand and address the problem. In Ethiopia, most veterinary antimicrobials are injectable, requiring repeated clinic visits or on-farm administration [9, 14]. Under these circumstances, follow-up observation using structured checklists represents a practical and context-specific method for assessing treatment compliance.

Despite growing concern about AMR, misuse of antimicrobials in Ethiopian livestock has been documented [9, 15], yet no published studies have assessed treatment compliance or predictors of non-compliance among animal owners. Previous work has focused on antimicrobial use and resistance patterns [9, 15, 16]. This study provides the first empirical evidence on compliance, identifying key drivers of non-compliance and proposing interventions. Treatment compliance has clear One Health implications: misuse not only affects animal health and productivity but also contributes to resistant pathogens that threaten human health and food safety [9, 17]. By filling this gap, the study contributes to antimicrobial stewardship strategies in Ethiopia. Improved compliance enhances clinical outcomes, supports preventive care, and promotes better animal welfare [2]. Building trust with veterinarians, acknowledging past treatment experiences, and strengthening communication and education about risks are key strategies to sustain compliance and promote antimicrobial stewardship [17, 18]. Therefore, this study designed to:

- Assess animal owners’ compliance with prescribed treatment regimens,
- Explore factors influencing their compliance
- Identify reasons for non-compliance and propose evidence-based interventions.

## Materials and methods

### Study area

This study was conducted at the University of Gondar Veterinary Teaching Hospital in Gondar town, the capital of the Central Gondar Zone, located at 12.6°N and 37.47°E with an elevation of about 2,133 m above sea level. Study animals originated from Gondar town and its surroundings. Gondar town has a livestock population of 78,123 cattle, 20,695 sheep, 21,515 goats, 9821 equids, and 17,280 dogs and cats [19].

According to the recorded data from the Veterinary Teaching Hospital, on average, in the hospital, 3,672 cattle, 5,052 sheep and goats, 4,014 horses, 27,832 donkeys, 1,896 dogs, and 132 cats have been treated annually for medical, surgical, gynecological, and preventive health conditions. Animal health service delivery in the area is primarily provided by government veterinary clinics, with limited participation from private practitioners. The University of Gondar Veterinary Teaching Hospital serves as the main referral and teaching facility, offering a wide range of services including treatment, vaccination, deworming, external parasite control, reproductive health management, and surgical operations [20].

### Study design and study population

A prospective observational study was conducted at the University of Gondar Veterinary Teaching Hospital from February to August 2025 to assess animal owners’ compliance with prescribed treatment regimens and to identify factors influencing their compliance. The study involved cattle, sheep, dogs, and horses that were presented to the University of Gondar Veterinary Teaching Hospital for the diagnosis and treatment of various disorders. Animal owners seeking treatment were drawn from both urban and rural areas of Gondar. Goats and other species were excluded due to their relatively lower case numbers during the study period.

### Sampling technique and sample size determination

Animal owners were selected based on the inclusion criteria. They were recruited when they brought their animals to the University of Gondar veterinary teaching hospital for treatment. Since no prior study had assessed treatment compliance among animal owners in Ethiopia, a prevalence of 50% was assumed to maximize sample size.

The approximate sample size was determined by considering a 50% expected prevalence of the problem with a defined precision of 10% and a level of confidence of 95%. Hence, the sample size was calculated as [21]:

$$\:\mathrm{n}=\frac{{\mathrm{Z}}^{2}\times\:\mathrm{p}\times\:\mathrm{q}}{{\mathrm{L}}^{2}}$$

Where:

n = required sample size

Z = confidence level at 95% (1.96)

p = expected prevalence (0.5, for maximum variability)

q = 1 – *p* = 0.5

L = margin of error (0.1 or 10%)

Thus, the minimum required sample size was 96 participants. To account for potential non-response or missing data, 10% was added, yielding a final sample size of 106 animal owners. Therefore, a total of 106 animal owners were enrolled in this study.

### Inclusion and exclusion criteria

Owners who had at least one animal prescribed antibiotic for a minimum of three consecutive days were included in the study. Those whose animals received treatment for fewer than three days were excluded, as shorter durations were considered insufficient for reliably assessing compliance using our criteria.

### Data collection methods and procedures

To assess animal owners’ compliance with a three-day antibiotic regimen at the University of Gondar Veterinary Teaching Hospital, a structured checklist was developed with reference to Ethiopia’s Standard Veterinary Treatment Guidelines [3], which recommend treatment durations ranging from one day to several weeks. Prior to the main study, the checklist was pre-tested on 5% of animal owners who were not included in the final sample. To evaluate the validity, follow-up observations were conducted by both the researcher and a non-clinician staff member using a study-specific checklist to document compliance events. Although the checklist was not a standardized instrument, its consistent application across repeated observations provided reasonable assurance of its utility in this context.

Owners whose animals were prescribed a three-day course of treatment were enrolled following clinical examination and diagnosis by attending clinicians, with treatment details obtained from prescription papers. Prior to treatment, owners were asked to participate and interviewed to collect demographic information. They were informed that the prescribed regimen would last three days; however, to reduce the likelihood that interviews influenced compliance behavior; owners were not told that compliance itself was being assessed. Animals then received treatment, and owners were instructed to return daily until the regimen was completed. Daily attendance was documented through direct observation using a structured follow-up checklist.

To monitor for non-compliance, researchers arrived at the hospital before the scheduled treatment time and observed whether owners attended. The antibiotics prescribed by clinicians during the study included Procaine Penicillin, Penstrep, sulfamethoxazole–trimethoprim, Oxytetracycline 10%, and ceftriaxone. The choice of antibiotics prescribed was left to clinicians, and no changes in drug type or treatment duration occurred during the study. Species treated, prescribed duration, and the number of follow-up observations were documented using the checklist.

Compliance was defined as completion of all three prescribed days, while non-compliance was measured directly by the number of doses missed. A total of 106 animal owners participated. Owners who missed the middle dose but returned for the final dose were interviewed face-to-face to explain their reasons, while those who missed both doses were contacted by phone. Semi-structured, open-ended questions were used to explore reasons for missed doses. During the study, no cases occurred where owners attended but animals failed to receive treatment, and no changes in drug type or treatment duration were observed.

### Data management and analysis

The collected data were first entered into Microsoft Excel and cleaned for accuracy. Subsequent analysis was conducted using the Statistical Package for Social Sciences (SPSS) version 20. Frequencies and percentages were used to summarize demographic characteristics, animal species, missed doses, reasons for non-compliance, and compliance distribution across demographic groups and animal species. Logistic regression analysis was employed to examine factors influencing owner compliance. The association was considered statistically significant at a *p*-value ≤ 0.05. First, univariable analysis was performed. Variables with *p* ≤ 0.25 were selected for the multivariable model.

Prior to this, the variance inflation factor (VIF) was tested to identify the presence of multicollinearity among independent variables; all VIFs were < 10, indicating no significant multicollinearity. A generalized linear model (GLM) with a binomial-logit link function was fit for the data. Model fit was assessed using the Hosmer-Lemeshow test (*p* > 0.05 indicating good fit) and the Omnibus test (*p* < 0.05 for significant predictors). A univariable analysis was first performed. Variables with *p* ≤ 0.25 were included in the multivariable model [9].

All variables were checked for missing values prior to analysis. No missing data were identified for the variables included in the multivariable model. Therefore, all participants were included in the final model. An interaction between owner age and prior experience with animal illness, farming experience and prior experience with animal illness, and education level between farming experience was tested. None of the interaction terms was statistically significant. Results from both univariable and multivariable logistic regression analyses were reported as odds ratios (OR) and Adjusted OR (aOR), respectively, together with their 95% confidence intervals (CIs) at a *p*-value ≤ 0.05.

## Results

### Demographic characteristics of participants

Most animal owners who participated in the study were aged between 30 and 40 years (47.2%), male (83.0%), and cannot read and write (50.0%). A small proportion (10.4%) of participants had attained tertiary-level education. Most participants (58.8%) had less than five years of experience in livestock farming or animal ownership. A small proportion (34.9%) had experience of owning multiple animals of different species, and 29.2% had experience of owning animals for the first time. About 39.6% of participants reported experience with illnesses only in the animal presented to the hospital (this animal), and 21.7% had prior experience with illnesses in other animals of the same species (Table 1).

**Table 1:** Demographic characteristics of animal owners’

Variables	Categories	*n* (%)
Age (years)	20–30	29 (27.4)
	30–40	50 (47.2)
	> 40	27 (25.5)
Gender	Female	18 (17.0)
	Male	88 (83.0)
Level of education	Cannot read and write	53 (50.0)
	Primary school	25 (23.6)
	Secondary school	17 (16.0)
	Tertiary	11 (10.4)
Farming experience (years)	> 10	62 (58.5)
	5–10	26 (24.5)
	< 5	18 (17.0)
Prior experience with animal ownership	First animal	31 (29.2)
	Multiple animals	37 (34.9)
	Multiple animals, multiple species	38 (34.9)
Prior experience with animal illness	Multiple species	41 (38.7)
	Other animals of the same species	23 (21.7)
	The animal under treatment	42 (39.6)
Total		**106 (100.0)**

*n* number, *% *percent, first animal: owner had experience of ownership to the animal that was under treatment, this animal: owner had experience in illness to the animal that was under treatment

### Compliance distribution across demographic characteristics and species of animal

The overall compliance rate was 68.9%. Higher compliance was observed among owners aged 20–30 years (75.9%), females (77.8%), and those with secondary education (76.5%). Cattle owners showed higher compliance (77.8%). Owners with 5–10 years of farming experience demonstrated the highest compliance (92.3%). most owners (76.3%) with prior experience of keeping multiple animals of multiple species were more compliant with prescribed treatment regimen. Compliance was also very high (91.3%) among owners who had previously managed illnesses in other animals of the same species as the one under treatment. A total of 33 owners (31.1%) did not follow the prescribed regimen. Non- compliance was particularly evident among those with less than five years of farming experience (55.6%), first-time animal owners (48.4%), and participants facing illness in their animals for the first time (50.0%) (Table 2).

**Table 2:** Compliance distribution across demographic characteristics and species of animal

Variables	Categories	Compliant (*n* (%))	Non-compliant (*n* (%))
Owner age (years)	20–30	22 (75.9)	7(24.1)
	30–40	36 (72.0)	14 (28.0)
	> 40	15 (55.6)	12 (44.4)
Owner gender	Female	14 (77.8)	4 (22.2)
	Male	59 (67.0)	29 (33.0)
Educational level	Primary	19 (76.0)	6 (24.0)
	Cannot read and write	33 (62.3)	20 (37.7)
	Secondary	13 (76.5)	4 (23.5)
	Tertiary	8 (72.7)	3 (27.3)
The species of animal presented to the Hospital	Sheep	18 (67.7)	9 (33.3)
	Cattle	21 (77.8)	6 (22.2)
	Dog	18 (69.2)	8 (30.8)
	Horse	16 (61.5)	10 (38.5)
Farming experience (years)	> 10	41 (66.1)	21 (33.9)
	5–10	24 (92.3)	2 (7.7)
	< 5	8 (44.4)	10 (55.6)
Prior experience with animal ownership	First animal	16 (51.6)	15 (48.4)
	Multiple animals	28 (75.7)	9 (24.3)
	Multiple animals, multiple species	29 (76.3)	9 (23.7)
Prior experience with animal illness	Multiple species	31 (75.6)	10 (24.4)
	Other animals of the same species	21 (91.3)	2 (8.7)
	The animal under treatment	21 (50.0)	21 (50.0)
Total (*N* = 106)		**73 (68.9)**	**33 (31.1)**

*n* number, *% *percent, first animal: owners had experience of ownership of the animal that was under treatment, this animal: owners had experience with the illness of the animal that was under treatment

**Fig. 1:**
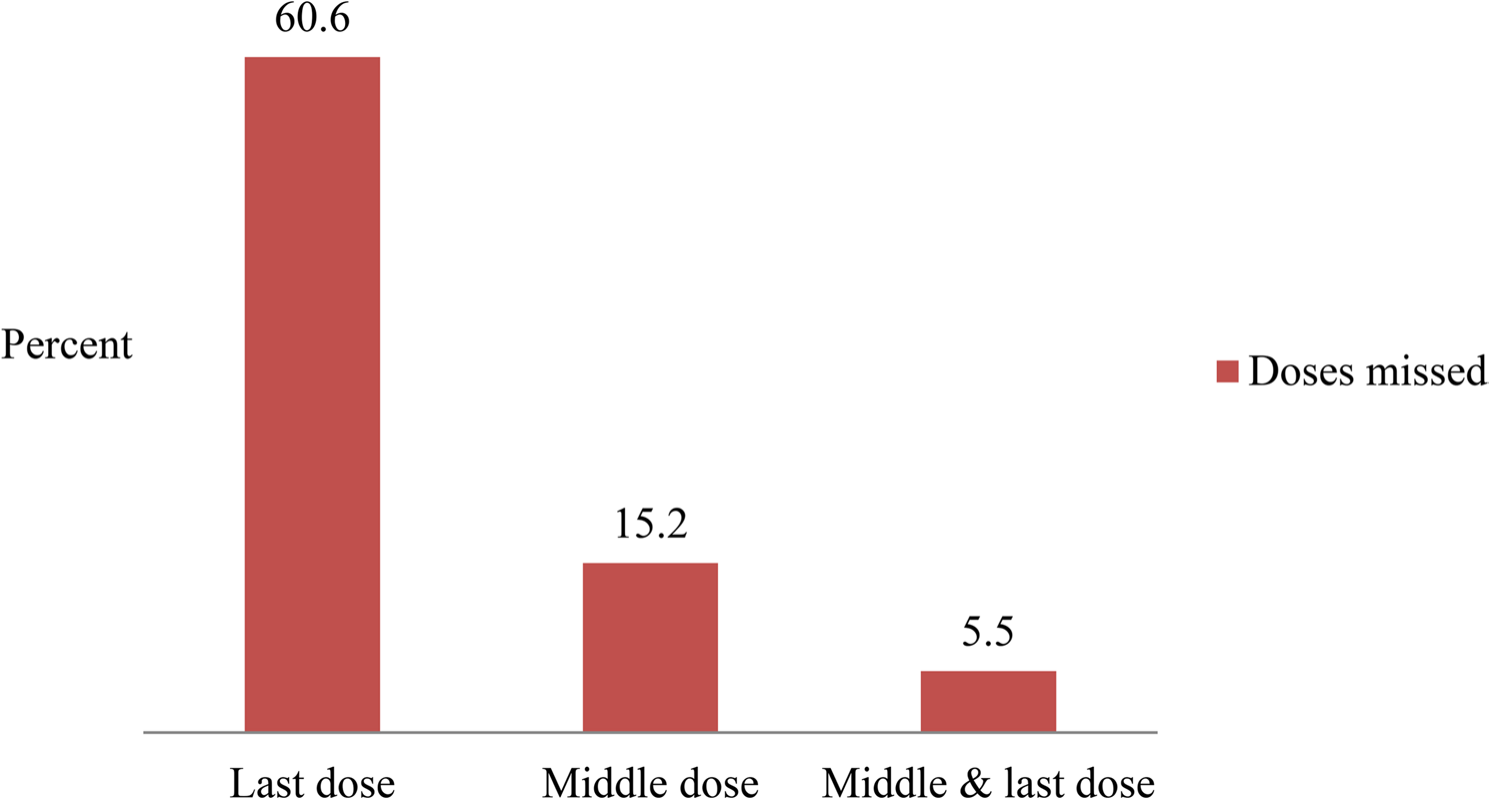
The doses missed among non-compliant owners. Last dose: dose to be given on the third day, middle dose: dose to be given on the second day, middle and last dose: dose to be given on the second and the third day

The number of times the treatment was missed by the owners was assessed. The last dose was most frequently missed (60.6%). Only a few owners (7.5%) missed the middle and last dose (Fig. 1).

**Fig. 2:**
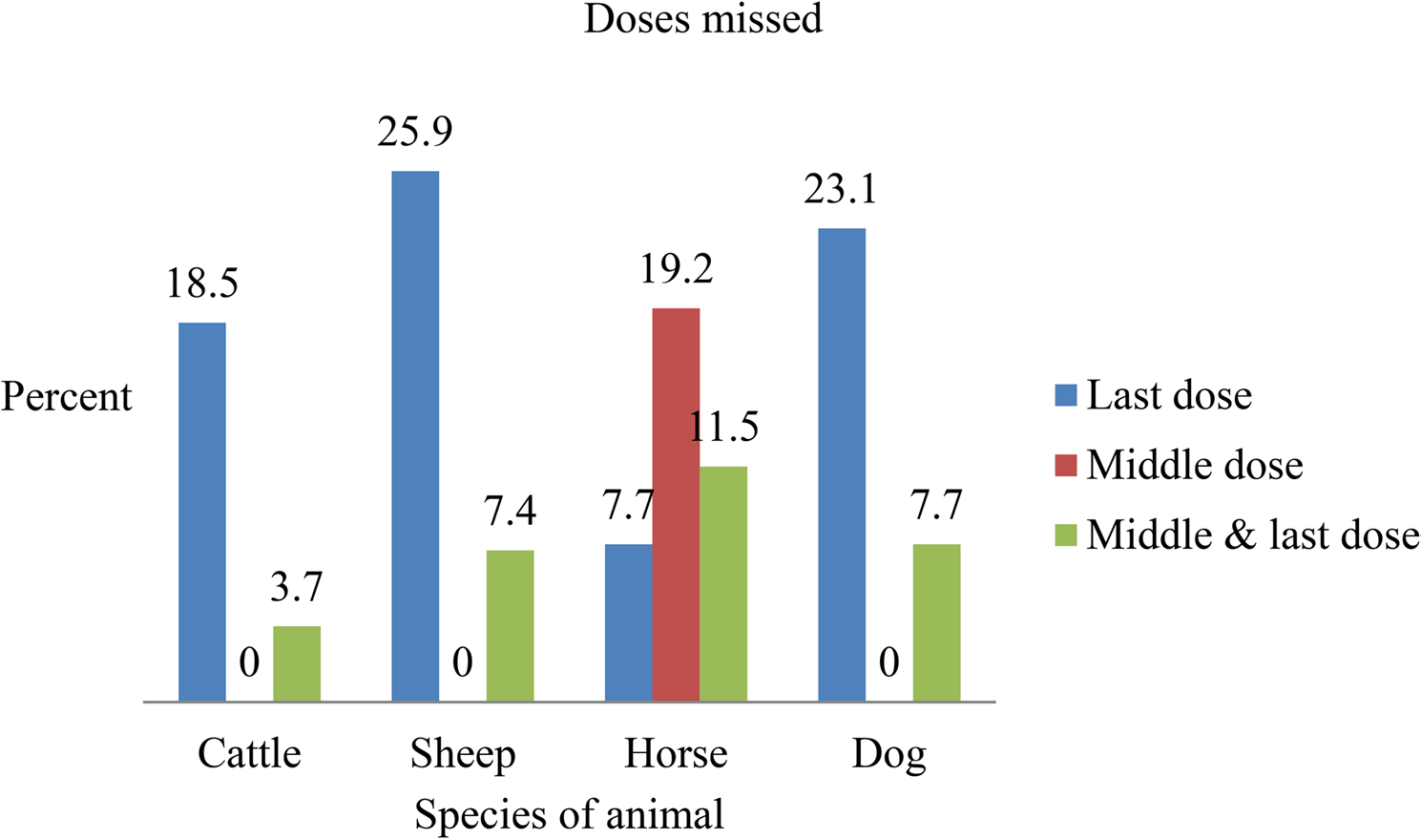
Frequency of the doses missed among different animals. Last dose: dose to be given on the third day, middle dose: dose to be given on the second day, middle and last dose: dose to be given on the second and the third day

The doses missed among animal species presented to the hospital were also assessed. The last dose was most frequently missed by sheep owners (25.9%). Horse owners missed two doses, in which 19.2% of horses didn’t receive the middle dose and 11.5% didn’t receive the middle and last doses (Fig. 2).

**Fig. 3:**
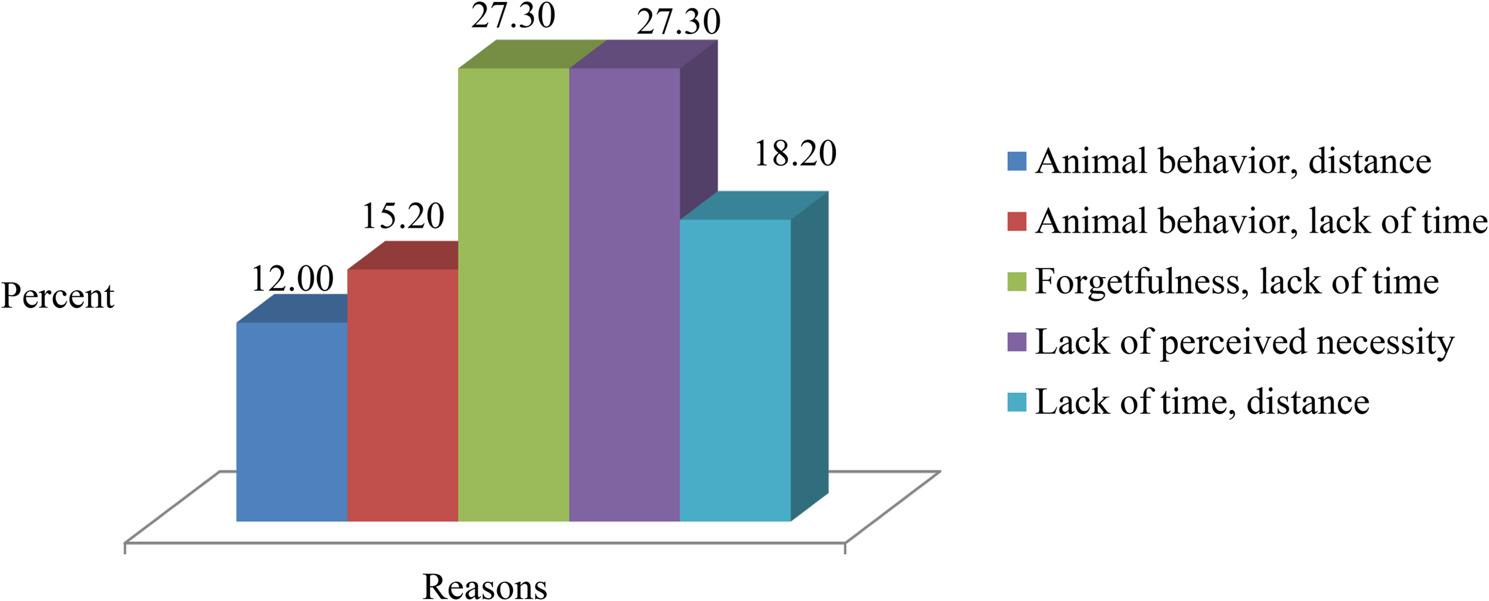
Reasons indicated by non-compliant owners for missing treatment

Animal owners’ reasons for treatment non-compliance were described in Fig. 3. The majority of animal owners mentioned that lack of perceived necessity (27.3%), forgetfulness, and lack of time (27.3%) were the main reasons for non-compliance. A smaller portion (12.0%) indicated that animal behavior and distance from the clinic were the reasons for missing treatment (Fig. 3).

Variables with a p-value ≤ 0.25 were selected for initial inclusion in the multivariable logistic regression model based on the results of univariable logistic regression analyses of associations between compliance and demographic factors (Table 3).

**Table 3:** Univariable logistic regression model for factors associated with owner compliance with the prescribed treatment regimen

Variables	Categories	B	OR	95% CI	*p*-value
Owners’ age (years)	20–30	0.92	2.51	20.80–7.86	0.113
	30–40	0.72	2.06	0.77–5.47	0.148
	> 40	Rf			
Owners gender	Female	0.54	1.72	0.52–5.69	0.374
	Male	Rf			
Educational level	Primary	0.65	1.92	0.66–5.61	0.234
	Secondary	0.68	1.97	0.56–6.88	0.288
	Tertiary	0.48	1.62	0.38–6.81	0.513
	Can’t read and write	Rf			
Animals presented to the Hospital	Cattle	0.56	1.75	0.52–5.87	0.365
	Dog	0.12	1.13	0.35–3.57	0.842
	Horse	-0.22	0.80	0.26–2.46	0.697
	Sheep	Rf			
Farming experience (year)	> 10	0.89	2.44	0.84–7.10	0.102
	5–10	2.71	15.00	2.69–8.45	0.002
	< 5	Rf			
Prior experience with animal ownership	Multiple animals, multiple species	0.04	1.04	0.36–2.99	0.948
	First animal	-1.07	0.34	0.12–0.96	0.042
	Multiple animals	Rf			
Prior experience with animal illness	Multiple species	-1.22	0.29	0.06–1.48	0.139
	Other animals of the same species	-2.35	0.09	0.02–0.46	0.003
	The animal under treatment	Rf			

*B* coefficient, *OR *odds ratio, *CI *confidence interval, *Rf *reference category, first animal: owner had experience of ownership to the animal that was under treatment

Although several variables showed associations in the univariable analysis (*p* ≤ 0.25), only education level, farming experience, and prior experience with animal illness remained significant in the multivariable model. In the multivariable analysis, the variable prior experience with animal illness was affected by an imbalance in participant numbers between the reference category (owners with experience in other animals of the same species, *n* = 23) and those with experience in the animal under treatment (*n* = 42). This uneven distribution resulted in wide confidence intervals, limiting the precision of the estimates.

Owners who attained primary and tertiary levels of education, respectively, were 6-and 7-times more likely (aOR = 6.526, 95% CI: 1.05–40.57, *p* = 0.044; aOR = 7.256, 95% CI: 1.17–44.91, *p* = 0.033) to be compliant than those who cannot read and write. Owners with five to ten years of farming experience were 13 times more compliant (aOR = 13.193, 95% CI: 1.97–88.46, *p* = 0.008) compared to those with less than five years of experience (Table 4). Owners who had first-time experience with animal illness (animal under treatment) were 0.029 times less likely to be compliant (aOR = 0.029, 95% CI: 0.00-0.35, *p* = 0.005) with the prescribed treatment regimen compared to those owners with prior experience of illness in other animals of the same species (Table 4).

**Table 4:** Multivariable logistic regression for factors associated with owner compliance with the prescribed treatment regimen

Variables	Categories	B	aOR	95% CI	*p*-value
Owners’ age (years)	20–30	0.747	2.110	0.34–13.04	0.422
	31–40	0.865	2.376	0.62–9.09	0.206
	> 40	Rf			
Educational level	Primary	1.876	6.526	1.05–40.57	0.044
	Secondary	0.934	2.545	0.44–14.78	0.298
	Tertiary	1.982	7.256	1.17–44.91	0.033
	Can’t read and write	Rf			
Farming experience (year)	> 10	1.044	2.840	0.55–14.71	0.214
	5–10	2.580	13.193	1.97–88.46	0.008
	< 5	Rf			
Prior experience with animal ownership	Multiple animals, multiple species	1.705	5.504	0.60-50.34	0.131
	First animal	0.644	1.905	0.28–13.06	0.512
	Multiple animals	Rf			
Prior experience with animal illness	Multiple species	-2.266	0.104	0.01–1.46	0.093
	The animal under treatment	-3.539	0.029	0.00-0.35	0.005
	Other animals of the same species	Rf			

*B* coefficient, *aOR *adjusted odds ratio, *CI *confidence interval, *Rf *reference category, first animal: owner had experience of ownership to the animal that was under treatment, this animal: owner had experience in illness to the animal that was under treatment

## Discussion

Non-compliance with the prescribed treatment regimens can result in treatment failure, the development of antimicrobial resistance, compromised animal welfare, economic losses, and adverse public health outcomes [1, 3]. This study assessed animal owners’ compliance with short-course injectable antibiotic regimens and identified key determinants of compliance. To the best of our knowledge, this is the first study to explore compliance patterns among animal owners in a setting where literacy is low and veterinary services are clinic-based, thereby addressing a critical gap and contributing to improved antimicrobial stewardship and One Health interventions.

The degree to which owners complied with the treatment regimen to administer a three-day course of antibiotics to their animal was assessed by direct observation using a follow-up checklist. In this study, four species of animals were included to assess owners’ compliance with the prescribed treatment regimen. Overall compliance was 68.9%, defined as completion of the full prescribed course. This level falls within the moderate compliance range (50–75%) commonly reported in human compliance studies [22]. The rate was higher than the 56% reported in owner compliance in canine epilepsy by Booth et al. [2], but lower than the 87.4% reported by Ribas et al. [23] Such discrepancy likely reflects differences in animal species, prescription duration, and assessment methods used [1, 2]. Nonetheless, these studies were included to provide context. While direct equivalence across species is limited, each assessed owner compliance with prescribed regimens, thereby offering a useful framework for situating our findings within the broader veterinary compliance literature.

Half of the animal owners (50.0%) reported being unable to read and write, while the other half had some level of formal education. This indicated the critical role of literacy in influencing compliance and suggesting veterinarian to undertake interventions to clarify the regimen and risk of missing treatment. Beyond overall compliance, several owner characteristics influenced adherence. Descriptive analysis indicated higher compliance among younger, female, and more educated owners, with 5–10 years of farming experience, prior experience of owning multiple animals of multiple species, and managing illnesses in multiple species were compliant, as well as cattle owners.

However, in the multivariable model, only education level, farming experience and prior experience remained significant independent predictors. Multivariable analysis revealed that education level, farming experience, and prior illness exposure were significant independent predictors of compliance.

Owners with primary (aOR = 6.526, *p* = 0.044) and tertiary (aOR = 7.256, *p* = 0.033) education were significantly more likely to comply compared with those who could not read or write. Education likely enhances awareness of the risks of non-compliance and builds trust in veterinary guidance, thereby improving compliance. Similarly, owners with five to ten years of farming experience had higher odds of compliance, being 13 times more likely to comply than those with less than five years of experience (aOR = 13.193, *p* = 0.008). Moderate farming experience appears to provide sufficient exposure to livestock management and veterinary services, reinforcing compliance, whereas very limited experience may hinder appreciation of veterinary advice [1, 24]. Taken together, these findings suggest that both formal education and practical farming experience improve compliance, while the lack of either reduces it compliance.

Prior experience with animal illness was associated with compliance. Owners encountering illness for the first time had significantly lower odds of adherence (aOR = 0.029, *p* = 0.005) compared to those with prior experience. However, the extremely low odds ratio and wide confidence interval (0.00–0.35) suggest instability due to sparse data, so this result should be interpreted cautiously as it may reflect a small sample size rather than a precise effect estimate. Even with this limitation, the pattern indicates that compliance may be influenced not only by education but also by perceived risk and memory of past outcomes. Prior experience appears to encourage adherence, whereas first-time encounters may reduce compliance through limited engagement with veterinary services and reduced appreciation of the importance of treatment [1].

Compliance also varied across animal species, reflecting economic and practical considerations. Cattle owners demonstrated the highest compliance (77.8%). Because cattle are directly tied to household income, food security, and farming efficiency, which may motivate compliance with the prescribed regimen [25]. In contrast, sheep and horse owners showed a greater drop-off, particularly at later doses.

Compliance declined progressively across the regimen, with a critical gap at the final stage, where 60.6% missed the last dose. Stopping early leaves residual infection, reduces treatment efficacy, and heightens the risk of relapse and antimicrobial resistance [13]. Among the last doses missed (25.9%), sheep owners accounted for the highest proportion, whereas horse owners showed a consistent drop-off across both the middle (19.2%) and final doses (11.5%). These findings suggest that compliance challenges vary by species and may reflect discontinuation once clinical signs improve or barriers such as time, distance, and animal behavior.

In addition to owner characteristics, practical and attitudinal barriers further shaped compliance. Owners cited lack of perceived necessity (27.3%), forgetfulness and time constraints (27.3%), animal behavior, and distance from the clinic (12.0%) as reasons for missed doses. These findings show that compliance is hindered by attitudinal barriers such as reliance on visible symptoms and practical barriers, including time, distance, and animal behavior. Such misconceptions pose a significant One Health concern, as premature discontinuation increases the risk of treatment failure and contributes to antimicrobial resistance [13]. These findings urge the need for targeted interventions, including strengthened veterinary-client communication about the risks of stopping regimen earlier, appropriate written instruction where possible, and reminder-based support systems where possible to improve compliance and promote responsible antimicrobial use in animals.

Although our study did not directly quantify treatment outcomes, as it was not designed as a clinical trial, our findings on compliance patterns underscore the critical importance of compliance. Missing doses undermines treatment success by prolonging illness and increasing relapse risk in animals [1, 12]. Furthermore, sub-therapeutic exposure fosters antimicrobial resistance, posing a significant One Health concern. Full compliance is therefore essential to optimize clinical outcomes, reduce treatment costs, and safeguard public health. Compliance with the prescribed regimen is essential for therapeutic success. Consistent compliance prevents relapse, limits antimicrobial resistance, and ensures effective treatment outcomes. In contrast, non-compliance reduces drug effectiveness, prolong illness, and increase the risk of recurrence in both humans and animals [12, 26].

The limitations of this study include the absence of follow-up data on animal health outcomes after missed doses, reliance on owner recall and sparse data in the variable ‘prior experience with animal illness. This prevented the assessment of the clinical consequences of non-compliance, may introduce potential bias and reduces generalizability, and limited the precision of the multivariable analysis by producing wide confidence intervals. This weakens confidence in the observed associations and constrains conclusions about the role of prior experience in compliance behavior. This confines our findings to patterns of owner compliance, rather than their impact on treatment effectiveness. Therefore, the compliance rates reported in this study should be interpreted as behavioral compliance measures, not as confirmed health outcomes.

## Conclusion

This study found a moderate overall compliance rate of 68.9% with prescribed treatment regimens. Compliance was significantly associated with education level, farming experience, and prior exposure to animal illness, with first-time owners markedly less likely to adhere. The main reasons for non-compliance were premature discontinuation after perceived recovery (27.3%) and forgetfulness or time constraints (27.3%). These findings indicate behavioral patterns that influence compliance and point to areas where veterinary support could be strengthened. To address this gap the followings are recommended:

- Provide counseling through veterinary extension services on the importance of treatment compliance and the risks of non-compliance, focusing on low-literacy and inexperienced owners, especially those who tend to stop treatment early after perceived recovery.
- Implement follow-up mechanisms such as SMS reminders and phone calls to reinforce treatment completion and sustain compliance.
- Conduct further research to assess animal health outcomes, quantify the impact of missed doses, and examine economic, access to veterinary services, and sociodemographic factors influencing compliance.

## Supplementary Information

Supplementary Material 1.

## Data Availability

Data is provided within the manuscript or supplementary information files.

## Abbreviations

AMR: Antimicrobial resistance
aOR: Adjusted odds ratio
CI: Confidence interval
GLM: A generalized linear model
SPSS: Statistical Package for Social Sciences
VIF: Variance inflation factor
