# Supplementary material for: Assessment of animal owners’ compliance with short-course antibiotic treatment at the University of Gondar Veterinary Teaching Hospital, Northwest Ethiopia
Source: BMC Vet Res. 2026 May 18;22:408. doi: 10.1186/s12917-026-05561-1 (PMC13352665; doi:10.1186/s12917-026-05561-1)
Supplement: Supplementary file 1 — Supplementary Material 1. [file 12917_2026_5561_MOESM1_ESM.docx]

Supplementary file S1

Questionnaire designed for follow-up study

Part I: Socio-Demographic profile of the Study Participants

1. Age of respondents:_______________________
2. Gender: a. Male b. Female
3. Current level of education
4. Primary B. Secondary c. Tertiary D. Can’t read and write
5. How many years of farming or ownership experience do you have:________________
6. Prior experience with animal ownership: A. first animal (animal that was presented to the clinic for treatment) b. multiple animals c. multiple animals, multiple species
7. Prior experience with animal illness: a. this animal (animal that was presented to clinic for treatment) b. multiple species c. other animals of the same species (for the animal that was presented to clinic for treatment)
8. Diagnosis:_____________
9. Drug prescribed:_________________, dose______ frequency of admistration:________
10. Additional questions for non-compliant owners: what could be the reason for missing the treatment? (Please describe):____________________________

Part 2: Follow-up checklist used to measure the compliance of the owner with the prescribed treatment regimen (tick on each box below number of observations).

| **S/N** | **Date** | **Animal species** | **Sex** | **Age** | **Code** | **Total duration (in day)** | **No. of observation** | | | Status of compliance |
| --- | --- | --- | --- | --- | --- | --- | --- | --- | --- | --- |
| 1 |  |  |  |  |  |  | 1 | 2 | 3 |  |
| 2 |  |  |  |  |  |  |  |  |  |  |
| 3 |  |  |  |  |  |  |  |  |  |  |
| 4 |  |  |  |  |  |  |  |  |  |  |
| 5 |  |  |  |  |  |  |  |  |  |  |
| 6 |  |  |  |  |  |  |  |  |  |  |
| 7 |  |  |  |  |  |  |  |  |  |  |
| 8 |  |  |  |  |  |  |  |  |  |  |
| 9 |  |  |  |  |  |  |  |  |  |  |
| 10 |  |  |  |  |  |  |  |  |  |  |
| 11 |  |  |  |  |  |  |  |  |  |  |
| 12 |  |  |  |  |  |  |  |  |  |  |
| 13 |  |  |  |  |  |  |  |  |  |  |
